# Pathogenesis of Cardiac Valvular Hemangiomas: A Case Report and Literature Review

**DOI:** 10.3390/ijms26157114

**Published:** 2025-07-23

**Authors:** Kimberly-Allisya Neeter, Catalin-Bogdan Satala, Daniela Mihalache, Alexandru-Stefan Neferu, Gabriela Patrichi, Carmen Elena Opris, Simona Gurzu

**Affiliations:** 1 Department of Pathology, Faculty of Medicine, ”George Emil Palade” University of Medicine, Pharmacy, Science and Technology, 540142 Targu-Mures, Romania; kim.neeter@gmail.com (K.-A.N.); gabrielaa.constantin@yahoo.com (G.P.); carmenchincisan@yahoo.com (C.E.O.); simona.gurzu@umfst.ro (S.G.); 2 Department of Pathology, Clinical County Emergency Hospital, 810325 Braila, Romania; daniela.mihalache@ugal.ro; 3 Department of Pathology, Faculty of Medicine, ”Dunarea de Jos” University, 800008 Galati, Romania; 4 Independent Researcher, 44791 Bochum, Germany; 5 Department of Pathology, Clinical County Emergency Hospital, 540136 Targu-Mures, Romania; 6 Department of Adult and Children Cardiovascular Recovery, Emergency Institute for Cardio-Vascular Diseases and Transplantation, 540136 Targu-Mures, Romania

**Keywords:** valvular hemangioma, arteriovenous hemangioma, cardiac tumor, tricuspid valve, valvular tumor, endothelial-to-mesenchymal transition, mesenchymal-to-endothelial transition, endocardial cushion

## Abstract

Valvular hemangiomas are uncommon vascular anomalies that appear on the surface of heart valves. They can cause an array of non-specific symptoms and are consequently rarely diagnosed, with only 31 such cases (including the present one) reported to date in the literature; the present case is the first report of an arteriovenous hemangioma with a tricuspid localization. During the preoperative echocardiographic examination for a ventricular septal defect, a mass was incidentally discovered on the tricuspid valve of a 9-month-old infant. The involved leaflet was surgically removed and sent to the pathology department for analysis and subsequently diagnosed as an arteriovenous hemangioma. The patient recovered well, with no local tumor recurrence or other complications. The microscopic examination showed multiple blood vessels which stained positive for the endothelial markers CD31 and CD34 and which did not express D2-40, normally found in lymphatic endothelia. Surprisingly, endothelial cells lining the vessels also showed positivity for SMA, a mesenchymal cell marker, indicating a possible involvement of endothelial-to-mesenchymal transition and its opposite process, mesenchymal-to-endothelial transition, in the pathogenesis of these vascular anomalies.

## 1. Introduction

Cardiac tumors are among the rarest neoplasms, with most cases diagnosed as secondary metastases that usually do not have a favorable evolution [1]. Primary tumors of the heart represent rarer entities than secondary tumors, and although they are predominantly benign in origin, they can prove to be a challenging diagnosis because of the non-specific symptoms they can exhibit [2].

According to the 2021 revised World Health Organization (WHO) Classification of Tumors of the Heart, papillary fibroelastomas are the most common benign primary cardiac tumors in adults, whereas rhabdomyomas and cardiac fibromas are most commonly reported in pediatric patients [3]. Hemangiomas, benign tumors of the blood vessels, rarely have a cardiac disposition and are therefore extremely hard to diagnose clinically. Moreover, similar to many cardiac tumors, hemangiomas can manifest with a variety of non-specific symptoms ranging from asymptomatic incidental discovery to rapidly declining cardiac function, based on their location, size and potential for embolization [1]. The histological variants of cardiac hemangioma included in the WHO classification are capillary, cavernous, venous, arteriovenous and NOS type (no specific type), but most of the information about these tumors comes from isolated case reports due to their rarity [3]. The valvular disposition of hemangiomas is even less frequent because the cardiac valves are considered avascular structures, except for the one-third of the proximal portion near the valve ring [4].

Endothelial-to-mesenchymal transition (EndMT) is a biological process in which endothelial cells lose their specific markers and acquire mesenchymal characteristics, contributing to fibrosis and vascular remodeling. Its counterpart, mesenchymal-to-endothelial transition (MEndoT), is associated with neovascularization. Both processes are thought to be involved in the pathogenesis of vascular tumors like hemangiomas, especially in atypically vascularized cardiac tissues [1,3].

In this paper, we present the case of an incidentally discovered arteriovenous tricuspid valve hemangioma in an infant. We also include a literature review of existing valvular hemangiomas, along with a discussion of molecular patterns that suggest possible correlations between endothelial-to-mesenchymal transition (EndMT) and its antithesis, mesenchymal-to-endothelial transition (MendoT), in the pathogenesis of vascular anomalies.

## 2. Case Presentation

### 2.1. Personal History

A 9-month-old infant was admitted to the Emergency Institute for Cardiovascular Diseases and Transplant Centre Targu Mures in August 2022 for faltering growth. The patient was diagnosed with patent foramen ovale and VSD at the age of three months, during a routine examination; appropriate treatment with diuretics, beta-blockers and angiotensin-converting-enzyme (ACE) inhibitors was commenced. Supervision and periodic cardiac evaluations were recommended, since there was no requirement for surgery at the time.

The evolution was not favorable; the mother of the child reported difficulties breastfeeding and the infant’s poor weight gain in the last six months. The exacerbation of the symptoms required surveillance and further investigations, for which the patient was hospitalized. No significant history of disease was reported in the patient or the rest of the family.

### 2.2. Clinical Assessment

During the general examination, the patient was weighed and measured, and the required growth charts were devised. The infant weighed 6 kg at the time of the examination, 2 kg less than the expected weight when compared to the ideal weight-to-age ratio established by WHO, showing a mild-to-moderate developmental deficit. The clinical assessment revealed a strawberry hemangioma on the patient’s forehead, along with pallor and craniofacial dysmorphism (triangular facial appearance, sharp chin and low-set ears), for which a genetic consultation was requested. Decreased muscle tone was also described.

Difficulty breathing was observed during the respiratory evaluation, highlighted by an increased respiratory rate (60 breaths/min; the normal rate for this age is 25–40 breaths/min) and intercostal retractions, but no abnormal findings during auscultation of the lungs were reported.

The cardiac examination revealed a fixed splitting of the second heart sound and a third-grade holosystolic murmur along the lower left sternal border, with no palpable thrill, both suggestive of VSD. An echocardiograph was requested for further evaluation.

### 2.3. Laboratory Results

Laboratory parameters were measured to evaluate the patient’s condition. Hematological findings included mild microcytic hypochromic anemia, with a hematocrit level of 32% (normal range: 33–38%) and a hemoglobin level of 10.3 g/dL (normal range: 10.5–13 g/dL), whereas the biochemistry results showed signs of malnutrition and tissue damage: hypoproteinemia (3.8 g/dL, normal range: 4.2–7.4 g/dL), increased lactate dehydrogenase/LDH (258 U/L, normal range: 110–144 U/L) and aspartate aminotransferase/AST (80 U/L, normal range: 18–74 U/L).

### 2.4. Imaging and Endoscopic Examinations

A chest X-ray and an echocardiographic examination were requested to assess the status of the patient.

The chest X-ray showed predominantly right-sided cardiomegaly, dilation of the pulmonary trunk and increased pulmonary vascularity, suggestive of congestive heart failure; the cardiothoracic ratio was 0.56 in the posteroanterior chest view. These findings were consistent with the diagnosis of heart failure secondary to VSD.

**Figure 1:**
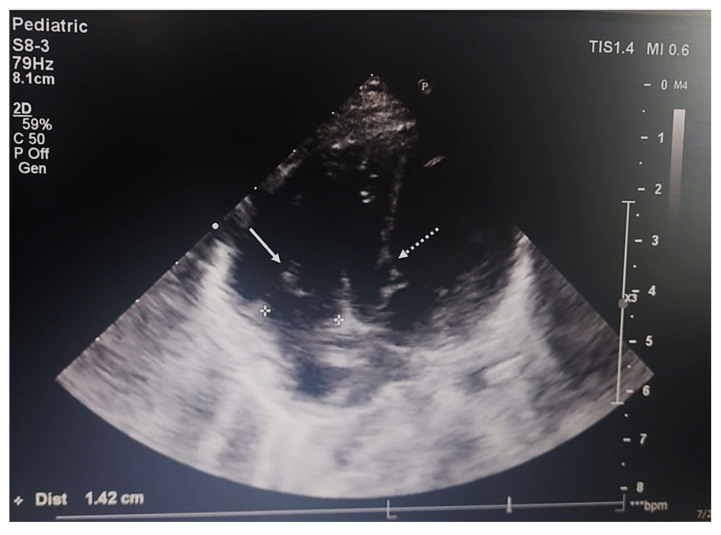
Preoperative transthoracic echocardiography, four-chamber view; the continuous arrow indicates a mass located on the tricuspid valve, which was diagnosed as an arteriovenous hemangioma; the dotted arrow shows a ventricular septal defect that was resolved surgically.

The echocardiogram showed dilation of the left atrium, but the patent foramen ovale diagnosed six months prior to this evaluation was no longer reported. The left ventricle and mitral and aortic valves presented no abnormalities. The right atrium and pulmonary artery were dilated, and a 5 × 3 mm circular mass was described on the posterior leaflet of the tricuspid valve. A mild tricuspid regurgitation was also reported, presumed to be secondary to both the right-sided cardiomegaly and the mass on the posterior leaflet of the valve. The pulmonary valve had a normal configuration. The interventricular septum presented a 6 mm discontinuity in the left ventricle outflow tract beneath the aortic valve, with a left-to-right shunt (pulmonary/systemic flow ratio = Qp/Qs = 2.92; Qp/Qs normal value = 1, left-to-right shunt > 1, right-to-left shunt < 1) (Figure 1). The defect was first diagnosed as a perimembranous ventricular septal defect in December 2021, and therapy with diuretics, beta-blockers and ACE inhibitors was initiated.

Taking into consideration the personal history and the clinical and paraclinical examinations, the final diagnosis was a perimembranous ventricular septal defect, a tricuspid valve mass and secondary ROSS III heart failure; the clinical status of the patient required surgical correction of the defect and removal of the valvular mass to improve cardiac function. The informed consent of the parents was obtained prior to the surgical intervention.

### 2.5. Surgery

Due to the location and size of the septal defect and the associated left-to-right shunt indicated by the pulmonary-to-systemic flow ratio, surgical closure was deemed necessary, concomitantly with excision of the mass located on the tricuspid valve. The VSD was resolved uneventfully using a heterologous pericardial patch, but the removal of the mass required complete resection of the posterior leaflet of the tricuspid valve. The Kay procedure was afterwards used to form a functional bicuspid valve composed of the remaining anterior and septal leaflets; this procedure involves suturing the area where the posterior leaflet is removed, allowing the remaining anterior and septal leaflets to function and form a competent bicuspid valve. The excised leaflet was then sent for histopathological assessment to the pathology department. The patient had an uneventful recovery and was discharged 9 days after surgery, with the following recommendations: genetic counselling for the facial dysmorphism and associated cardiac anomalies, endocarditis prophylaxis, and ferum supplements for the hypochromic microcytic anemia.

### 2.6. Gross and Histopathological Assessment of Surgical Specimens

The valve sample sent to the pathology department presented no significant findings apart from a relatively well-defined 4 × 3 × 3 mm, dark-colored mass attached to the midportion of the leaflet. The elastic mass had a smooth surface and a homogenous aspect on cut surface view.

**Figure 2:**
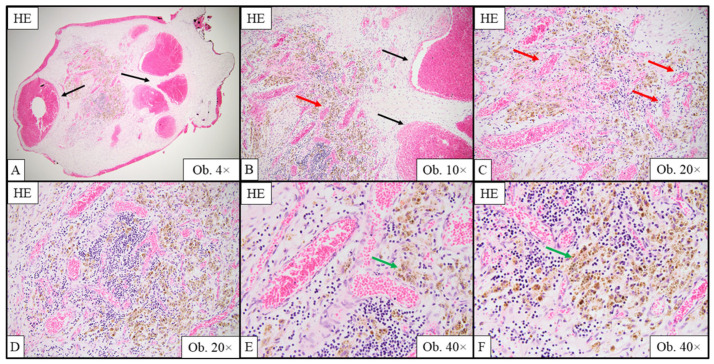
Histological features of the tricuspid valve hemangioma, emphasized with hematoxylin–eosin (HE). Large vascular spaces (**A**,**B**; black arrows) and smaller vessels (**C**,**D**; red arrows) containing red blood cells and surrounded by connective tissue can be observed; hemosiderin-containing macrophages, along with a mild inflammatory infiltrate, can be seen in the surrounding stroma (**E**,**F**; green arrows).

The histological examination with hematoxylin–eosin (HE) staining showed multiple, variably sized vascular spaces containing red blood cells, surrounded by connective tissue with a myxoid aspect (Figure 2).

**Figure 3:**
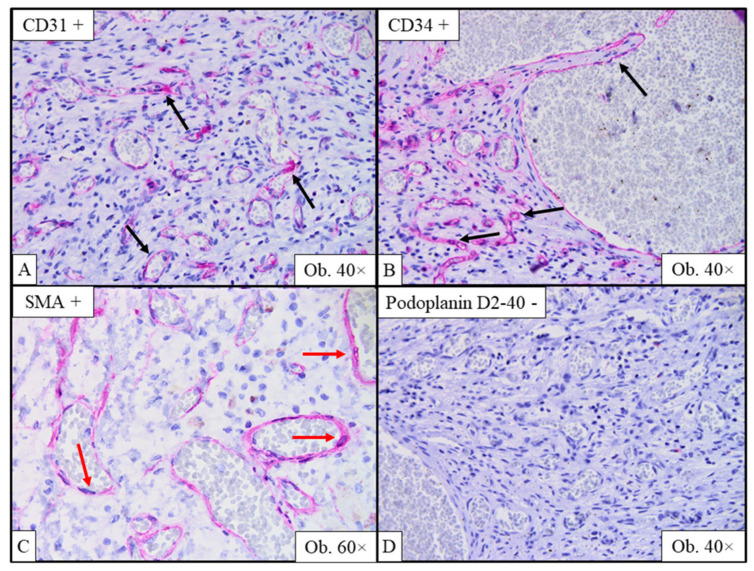
Immunohistochemical profile confirming the diagnosis of arteriovenous hemangioma. Positivity for CD31 (**A**; black arrows) and CD34 (**B**; black arrows) is specific to endothelial cells lining the vascular channels; paradoxically, endothelial cells stain positive for SMA, a mesenchymal cell marker (**C**; red arrows); podoplanin D2-40, negative in our case (**D**), is used to demonstrate the presence of lymphatic endothelium.

The vascular spaces were lined with a single layer of endothelial cells with no atypia, which stained positive for CD31 and CD34 (endothelial markers) and negative for podoplanin D2-40 (a lymphatic endothelium and mesothelium marker). Some vascular channels resembled veins; others had a well-represented SMA-positive muscular layer (smooth muscle actin) suggestive of arterial origin. Surprisingly, endothelial cells also showed positivity for SMA, a mesenchymal cell marker (Figure 3). Following the histopathological assessment, the mass was diagnosed as an arteriovenous hemangioma.

### 2.7. Outcome and Follow-Up

The patient recovered completely, and at the last periodic follow-up in 2024 the echocardiogram showed no tumor recurrence and no persistent VSD or left-to-right cardiac shunting; the tricuspid regurgitation was no longer described during examination; genetic counselling has not yet yielded any satisfactory results.

## 3. Discussion

Cardiac hemangiomas represent extremely rare entities, and their valvular extension is even more exceptional, with only 31 such tumors having been reported in the Medline database until February 2025, including the present case: 18 hemangiomas located on the mitral valve, 10 on the tricuspid valve and 3 on the aortic leaflets, and no pulmonary valve hemangiomas (Table 1) [5,6,7,8,9,10,11,12,13,14,15,16,17,18,19,20,21,22,23,24,25,26,27,28,29,30,31,32,33]. The most frequently diagnosed histological subtype is cavernous (15 cases) [5,6,7,10,13,16,17,18,21,22,27,28,29,31], followed by capillary (4 cases) [11,30,31,33] and arteriovenous types (2 cases) [25]; 1 case of combined capillary–cavernous hemangioma was reported [23], and 1 epithelioid hemangioma was described [15], but this subtype is not included in the WHO Classification of Heart Tumors [3]; in 8 cases, no histopathological diagnoses were included in the reports [8,9,12,14,19,20,24,26]. The average age at which hemangiomas were diagnosed was 43 years, with seven cases identified in children under the age of 10 [7,8,10,13,22,25] and two cases with no age specification. A slight female-to-male predominance was observed (15 to 13, excluding 3 cases where the patient’s sex was not mentioned), and the size of the hemangiomas ranged from 0.1 to 3 cm, with a mean of 1.72 cm. The correlated symptoms were added in the review, but some patients presented multiple pathologies, and the clinical manifestations might not have been directly caused by valvular hemangiomas (Table 1).

There is no universally accepted mechanism to explain the pathogenesis of cardiac hemangiomas, as the exact definition of these vascular anomalies is still disputed and the existing knowledge is limited to conclusions drawn from isolated case reports. According to the 2018 ISSVA Classification of Vascular Anomalies and Molecular Biology, vascular lesions should be divided based on the cells’ potential for autonomous proliferation into tumors and malformations [34]. Hemangiomas can be included in either classification, based on the histological subtype and mitotic activity [35]; however, little information exists about cardiac hemangiomas, and there is even less about valvular hemangiomas and how they should be regarded. Moreover, the evolution of hemangiomas can range between spontaneous regression and rapid proliferation, through mechanisms incompletely elucidated, making it harder to include them in one category [36,37]. The latest WHO Classification describes cardiac hemangiomas as either vascular tumors or malformations, with no distinction between the two [3]. A clear understanding of the underlying cause and mechanisms of cardiac hemangiomas, particularly valvular hemangiomas, could help define how these lesions should be classified. We think that endothelial-to-mesenchymal transition (EndMT) and its antithesis, mesenchymal-to-endothelial transition (MEndoT), processes intricately involved in heart development, might play a substantial role in valvulopathies, both congenital and acquired, including tumor growth at this site [38,39].

EndMT and MEndoT occur multiple times during cardiac development at the site of the future heart valves (the atrioventricular canal and outflow tracts), leading to endocardial cushion formation. This process begins when signaling from the myocardium triggers, through complex molecular cascades, a localized transformation of the adjacent endocardial endothelial cells into mesenchymal cells (EndMT cells) [40,41]. EndMT errors encountered during this stage of cardiac development lead to both valvular abnormalities and septal discontinuities, both of which were encountered in our case report [38]. The ability to induce EndMT is normally restricted to the valve-forming regions of the myocardium, but all of the endothelial cells lining the cardiac chambers have the ability to undergo EndMT if proper extrinsic signaling is present [42]. Endocardial/endothelial cell plasticity, meaning the cells’ ability to change between different cellular states, is maintained throughout adult life, a phenomenon necessary for both pathological and normal physiological processes [39]. In summary, aberrant EndMT and MEndoT could lead to vascularization of certain cardiac territories both during cardiogenesis and after birth, if relevant triggering signals are present, and could prove to be the mechanisms responsible for cardiac hemangiomas.

It is broadly recognized that epithelial-to-mesenchymal transition (EMT) and mesenchymal-to-epithelial transition (MET) also play major roles in cancer metastasis, but less is known about the involvement of EndMT and MEndoT in tumor progression. Reports of EndMT markers exhibited on the surface of vascular anomalies have drawn our attention towards EndMT as a possible causative mechanism for valvular hemangiomas [43,44,45]; the endocardial endothelial cells’ *SMA* expression in our case report supports this claim, together with the presence of septal defects associated with irregular EndMT [38].

In order to assess the molecular pathways involved in the appearance of hemangiomas, we selected six genes that are known to be of interest in relation to this subject and devised a complex network highlighting the interactions between them using the miRNet platform [46]. The following genes, already known to play a pivotal role in vasculogenesis, according to multiple studies, were included in the network: *TGF-β1*, *TGF-β2*, *NOTCH1*, *MAPK14*, *WNT1* and *PIK3CA* [34,40,47,48,49,50,51,52,53,54,55,56,57,58,59]. There are additional genes which have implications in this area of research, but the ones mentioned before are highlighted in many other studies and therefore represent the primary focus of our research.

**Figure 4:**
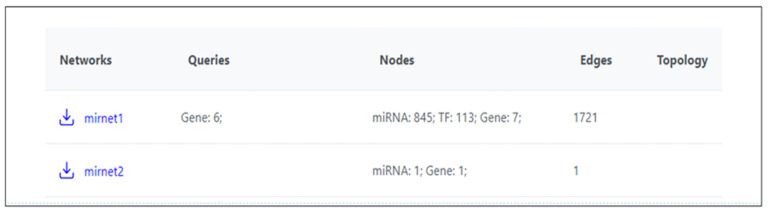
An input of six genes in the miRNet search engine revealed numerous interactions between them and various transcription factors and microRNAs. In total, 845 microRNAs and 113 transcription factors (TF) have resulted following this search, adding to a total of 1721 edges.

Following this search, a total of 845 microRNAs and 113 transcription factors of note have been identified (Figure 4).

**Figure 5:**
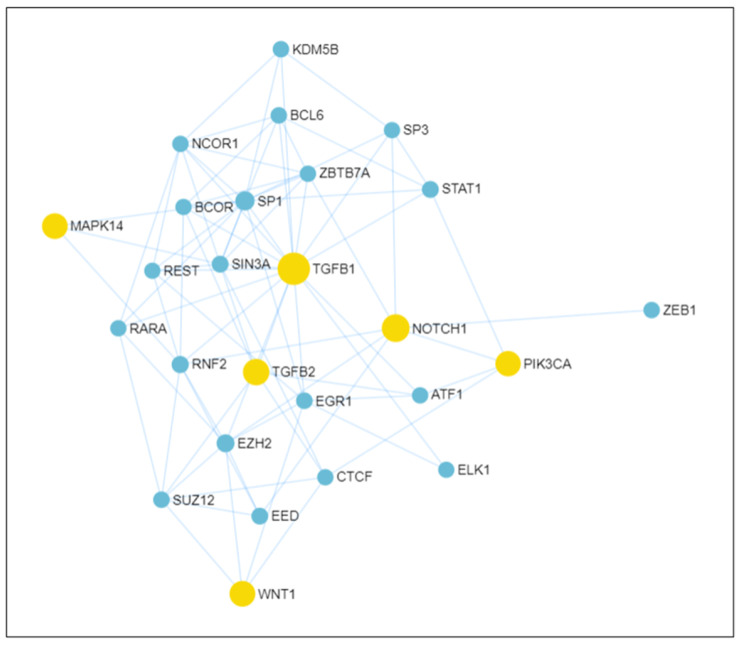
The major molecular pathways involved in EndMT, depicting the main genes involved; genes communicate through a complex network of micro-RNAs and transcription factors to transmit signals which influence EndMT. This pattern, showing the interactions between genes (yellow) and transcription factors (blue), was generated using the miRNet platform that collects information from different databases (miRTarBase v8.0, TarBase v8.0 and miRecords).

From all of these, we selected the most significant ones using the betweenness filter available on the platform. This process resulted in 20 transcription factors (TFs) that were selected and incorporated in the resulting network [48,49,50,51,53,54,59,60]: *ELK1*, *ZEB1*, *STAT1*, *BCL6*, *ATF1*, *REST*, *EGR1*, *EZH2*, *KDM58*, *NCOR1*, *BCOR*, *RARA*, *RNF2*, *CTCF*, *EED*, *SUZ12*, *SP1*, *SP3*, *ZBTB7A* and *SIN3A* (Figure 5).

It is also worth mentioning that all the input genes take part in essential molecular signaling pathways during cardiac development, specifically the EndMT process at the site of the endocardial cushions [39,47,60]. Errors encountered during this step in valve formation can lead to various cardiac anomalies [61]. Moreover, components of these molecular cascades persist even after the valves are formed, and their reactivation has been observed in diseased valves, suggesting an extensive correlation between this process and the pathophysiology of valvular diseases [38,47].

In regard to the mechanisms that partake in this transition, many intertwined molecular cascades participate in the EndMT process, but the leading contributors are the *TGF-β*, *Notch* and *Wnt* pathways, along with chain reactions activated by various growth factors (Figure 5) [47]. Of these, *PI3K/Akt* and *MAPK/ERK* are especially worth mentioning, since these pathways are mainly responsible for the formation of vascular anomalies [34]. Local environmental factors, such as inflammation, hypoxia and hyperglycemia, also contribute to EndMT induction [48].

Normally, EndMT is initiated when signaling from the canonical *TGF-β* pathway is transmitted through *BMP*s (bone morphogenetic proteins) to the endocardium, or non-canonically through alternative pathways such as *MAPK* and *PIK3CA/AKT* [47]. Afterwards, a synergy between the *Notch* and *TGF-β* pathways regulates and controls the transition of endothelial cells into mesenchymal cells, using both *BMPs*, agents of the *TGF-β* pathway, and *Jag*, operatives of *Notch* [60].

Notch signaling has various functions during embryonic development, including angiogenesis [47]; complete loss of *NOTCH1*, one of the four possible *Notch* receptors found primarily in the cardiac cushions, leads to disturbances in vascularization and angiogenetic defects [53,61]. It has not been tested whether overexpression of *NOTCH1* or hyperactivation of the Notch pathway can lead to excessive vascular formation, which could prove to be the cause of neovascularization in cardiac valves. Loss of *NOTCH1* also leads to a decrease in *SNAIL*, a known component of the EndMT/MEndoT process, suggesting that an overdrive mutation at this site could lead to an increased transition of mesenchymal cells into endothelial cells [62]; this aspect could be interpreted as a vascular malformation or tumor, and, ergo, a hemangioma. Moreover, the underexpression of this gene also leads to inadequate ventricular trabeculation, which results in various septal defects, as depicted in our case presentation [60]. Another possible cause of aberrant vascularization could be the production of proinflammatory molecules by the mesenchymal cells present in the normal heart valves following EndMT, similar to the inflammatory cytokines produced by the myofibroblasts in atherosclerosis [38,63].

There are many other molecular cascades which are connected through various transcription factors with the ones that regulate EndMT/MEndoT processes, such as *MAPK/ERK* and *PIK3CA/Akt/mTOR* [47]. According to the latest ISSVA Classification, the causative genes for vascular anomalies can be found in the same *MAPK/ERK* and *PI3K/AKT/mTOR* biological cascades [34]. These pathways are also intertwined with each other and with the Notch pathway, through the *RNF2* transcription factor, as can be seen in Figure 5. This transcription factor is a known repressor of *E-cadherin* transcription and, therefore, a promoter of EMT, as reported in a study on the implications of *RNF2* activity in the metastatic process [64]. Its involvement in this phenotypic transition is also suggested by our network (Figure 5).

Another possible communication point between the latter two pathways and the signaling cascades of EndMT and MEndoT is through the *SP1/STAT1* pathway, which connects them to the *TGF-β1* gene. *STAT1* is normally activated in response to inflammatory molecules, such as cytokines and growth factors, but it is believed that its effect on gene transcription is dependent on the synergic interaction with the *SP1* transcription factor [65]. Through signals received from proinflammatory cytokines, *STAT1* upregulates the activity of *ICAM-1*, a molecule with implications in both the immune system and cancer spread and metastasis [66]. Increased *ICAM-1* levels have also been reported in vascular inflammation phenomena present in atherosclerosis, myocardial infarction and other cardiovascular diseases [67]. Moreover, experimental metastatic tumor cells exposed to high levels of *ICAM-1* manifested an enhanced ability to secrete proangiogenic factors, such as *VEGF* and *PGE2* [68]. Therefore, the *SP1/STAT1/ICAM-1* pathway should be analyzed to establish its implications in the development of vascular neoplasms.

The *PIK3CA* cascade is also closely linked directly to the *WNT1* gene through a node formed by the *CTCF* transcription factor. The analysis of *CTCF* expression in different tumoral processes has led to the conclusion that this factor is a tumor-suppressing agent with a specific “change-of-function” activity, involved in cell differentiation [69]; its possible implications in EndMT/MEndoT and hemangiomas have not yet been studied.

Normally, upon their resolution, EndMT and MEndoT are kept inactive through transcriptional repressors, such as *ELK1*, the loss of which raises the barrier and enables the initiation sequence to take place. *ELK1* can be repressed by *mir-27b* upon signaling transmitted through the *TGF-β* pathway, thereby activating the transition process [39].

Even though this paper focuses on the molecular mechanisms potentially involved in cardiac carcinogenesis, it is important to emphasize that this is a preliminary hypothesis and requires further confirmatory studies. Moreover, signaling pathways both modulate and are shaped by the tumor microenvironment, suggesting even more complex interactions and further limiting the extent to which current research can fully explain this phenomenon [70,71].

**Figure 6:**
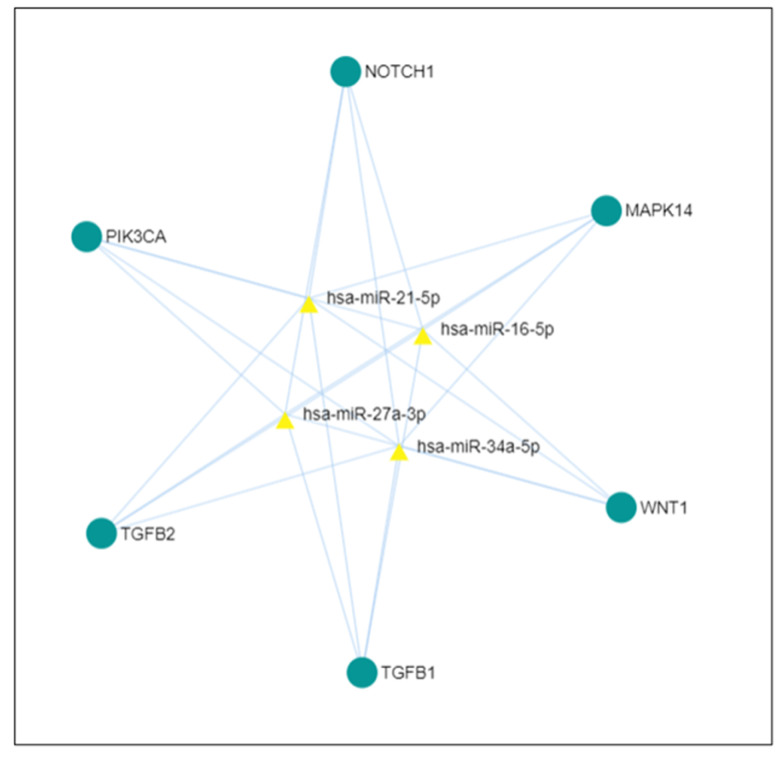
The microRNAs regulating the genes involved in both cardiovascular development and EndMT induction represented in a network created with the help of the miRNet platform, which gathers information from various databases. Four microRNAs of note (yellow) and the genes (green) that they regulate are depicted in this figure.

With the help of the miRNet platform, we have generated an additional network containing the most significant microRNAs that regulate the aforementioned genes’ expression [55,62,65,66,70] (Figure 6). MicroRNAs are non-coding sequences that regulate gene control at a post-transcriptional level. Four such transcripts are considered to be of importance for our research: *hsa-miR-16-5p*, *hsa-miR-34a-5p*, *hsa-miR-21-5p* and *hsa-miR-27a-3p*.

*Hsa-miR-16-5p* involvement has been studied in many malignancies since evidence of its activity was first reported in chronic lymphocytic leukemia [72]. Research has concluded that this microRNA has suppressive effects on the development of most malignancies, with the exception of ovarian tumors [72]. Moreover, when irregular activity of *hsa-miR-16-5p* has been detected, many cases have also shown errors in the PIK3CA/Akt signaling route, involved in vascular malformations, and NF-κB, a pathway necessary for the endothelial-to-mesenchymal transition [73].

Similar to the previous microRNA, *hsa-miR-34a-5p* has suppressor effects on carcinogenesis [74]. This area of study and the importance it might have regarding the molecular cascades that are of interest to our research are still underdeveloped and require further investigations.

In contrast to the carcinogenic-suppressing abilities of the previously mentioned miRNAs, *hsa-mir-21-5p* is a typical onco-miRNA, as suggested by its increased levels in the sera of patients with a wide range of cancers (pancreatic, breast, colorectal, brain, etc.) [75]. High expression of this miRNA is associated with a negative prognosis [76]; apart from its pro-carcinogenic activity, increased function of *hsa-miR-21-5p* has also been reported in the cardiovascular system, specifically in proliferative vascular disease, aortic valve calcification and cardiac hypertrophy, where it influences smooth muscle cell proliferation and cardiac fibroblast function [75].

*Hsa-miR-27a-3p*, the last remaining miRNA of note in our case, modulates the expression of *PDE3A*, an enzyme that maintains the normal function of the endothelial cells [77]; in the cardiovascular system, this microRNA plays essential roles in endothelial cell migration and angiogenesis [78].

## 4. Conclusions

The molecular pathways involved in EndMT are intertwined and strongly regulate one another, making it difficult to form a unified hypothesis as to what the causative flaw responsible for the septal atrioventricular defect and valvular hemangioma was in the case reported here. The rarity of the diagnosis makes it even harder to establish the exact cause; however, a link between aberrant EndMT/MEndoT and valvular hemangiomas exists, as highlighted by the expression of mesenchymal markers by the endothelial cells lining the vascular lumens of the tumor. It is important to emphasize the hypothetical nature of our research, but the putative role of EndMT and MEndoT in valvular hemangiomas should be taken into consideration and analyzed for a better understanding of vascular anomalies.

## Figures and Tables

**Table 1:** Cases of valvular hemangiomas reported to date (February 2025) in the Medline database.

Case No.	Authors, Year of Publication	Patient’s Gender	Patient’s Age (Years)	Valvular Localization	Histological Variant	Hemangioma Dimension (cm)	Clinical Manifestation
1.	Nye et al. 2001 [5]	Female	33	Mitral	Cavernous	1	Chest tightness, palpitations
2.	Lapenna et al. 2003 [6]	Female	49	Tricuspid	Cavernous	3	Syncope
3.	Ray et al. 2004 [7]	Male	47	Tricuspid	Cavernous	0.8	Dyspnea
4.		Male	0	Tricuspid	Cavernous	0.1–0.4 (multiple tumors)	Incidental detection during autopsy
5.	Wong et al. 2004 [8]	Female	4	Tricuspid	-	0.8	Cyanosis, systolic murmur
6.	Ahmed et al. 2004 [9]	-	-	Mitral	-	-	-
7.	Uğraş and Bayram 2005 [10]	Male	8	Mitral	Cavernous	3	Dyspnea, palpitations
8.	Vivirito et al. 2006 [11]	Male	81	Aortic	Capillary	-	Dyspnea, fever, thoracic pain
9.	Val-Bernal et al. 2006 [12]	Male	62	Aortic	-	3	Syncope, dyspnea
10.	Kutay et al. 2006 [13]	Male	8	Mitral	Cavernous	3	Dyspnea, palpitations, systolic murmur
11.	Muzzi et al. 2007 [14]	-	-	Mitral	-	-	-
12.	Dod et al. 2008 [15]	Female	86	Mitral	Epithelioid	2.4	Edema of lower extremities
13.	Yaganti et al. 2009 [16]	Female	33	Mitral	Cavernous	2	Chest pressure, dyspnea, lightheadedness
14.	Cannata et al. 2010 [17]	Male	76	Tricuspid	Cavernous	4	Incidental detection during echocardiographic evaluation
15.	Floria et al. 2011 [18]	Female	52	Tricuspid	Cavernous	1.6	Chest pain, palpitations
16.	Cook et al. 2011 [19]	Male	17	Mitral	-	1.4	Syncope
17.	Juric et al. 2013 [20]	Female	49	Mitral	-	0.9	Chest tightness, dyspnea
18.	Isbitan et al. 2014 [21]	Male	48	Mitral	Cavernous	-	Chest pain
19.	Gupta et al. 2016 [22]	Female	0	Tricuspid	Cavernous	0.8	Incidental detection during autopsy
20.	Val-Bernal et al. 2017 [23]	Male	62	Mitral	Capillary–cavernous	1.3	Edema of lower extremities
21.	Castelein et al. 2019 [24]	Female	60	Mitral	-	1.3	Incidental detection during echocardiographic evaluation
22.	Perez-Brandão et al. 2019 [25]	-	1	Mitral	Arteriovenous	0.75	Heart murmur
23.	Van Broekhoven et al. 2019 [26]	Male	74	Aortic	-	-	Aortic valve insufficiency, diastolic murmur
24.	Parkash et al. 2021 [27]	Female	78	Mitral	Cavernous	1 0.5 (two concomitant lesions)	Multifocal embolic brain infarcts
25.	Bayfield et al. 2022 [28]	Female	44	Mitral	Cavernous	3	Mitral regurgitation
26.	Toscano et al. 2022 [29]	Female	66	Mitral	Cavernous	2.8	Acute heart failure, myocardial infarction
27.	Ranjan et al. 2023 [30]	Male	52	Tricuspid	Capillary	1	Dyspnea, edema of lower extremities
28.	Kesieme et al. 2023 [31]	Female	49	Tricuspid	Cavernous	-	Dizziness, palpitations, syncope
29.	Osada et al. 2023 [32]	Female	56	Mitral	Capillary	1	Incidental detection during echocardiographic evaluation
30.	Rocco et al. 2024 [33]	Male	78	Mitral	Capillary	0.6	Incidental detection during echocardiographic evaluation
31.	Index case 2025	Female	0	Tricuspid	Arteriovenous	0.5	Incidental detection during echocardiographic evaluation

## Data Availability

The data used and reported in the Discussion section was generated using the online free-access miRNet platform: https://www.mirnet.ca/ (accesed on 15 January 2025).
